# Conjunctival MicroRNA Expression in Inflammatory Trachomatous Scarring

**DOI:** 10.1371/journal.pntd.0002117

**Published:** 2013-03-14

**Authors:** Tamsyn Derrick, Chrissy h. Roberts, Megha Rajasekhar, Sarah E. Burr, Hassan Joof, Pateh Makalo, Robin L. Bailey, David C. W. Mabey, Matthew J. Burton, Martin J. Holland

**Affiliations:** 1 Department of Clinical Research, Faculty of Infectious Tropical Diseases, London School of Hygiene and Tropical Medicine, London, United Kingdom; 2 Medical Research Council Unit, The Gambia, Fajara, Banjul, The Gambia, West Africa; 3 International Centre for Eye Health, Department of Clinical Research, Faculty of Infectious Tropical Diseases, London School of Hygiene and Tropical Medicine, London, United Kingdom; University of California San Diego School of Medicine, United States of America

## Abstract

**Purpose:**

Trachoma is a fibrotic disease of the conjunctiva initiated by *Chlamydia trachomatis* infection. This blinding disease affects over 40 million people worldwide yet the mechanisms underlying its pathogenesis remain poorly understood. We have investigated host microRNA (miR) expression in health (N) and disease (conjunctival scarring with (TSI) and without (TS) inflammation) to determine if these epigenetic differences are associated with pathology.

**Methods:**

We collected two independent samples of human conjunctival swab specimens from individuals living in The Gambia (n = 63 & 194). miR was extracted, and we investigated the expression of 754 miR in the first sample of 63 specimens (23 N, 17 TS, 23 TSI) using Taqman qPCR array human miRNA genecards. Network and pathway analysis was performed on this dataset. Seven miR that were significantly differentially expressed between different phenotypic groups were then selected for validation by qPCR in the second sample of 194 specimens (93 N, 74 TS, 22 TSI).

**Results:**

Array screening revealed differential expression of 82 miR between N, TS and TSI phenotypes (fold change >3, p<0.05). Predicted mRNA targets of these miR were enriched in pathways involved in fibrosis and epithelial cell differentiation. Two miR were confirmed as being differentially expressed upon validation by qPCR. miR-147b is significantly up-regulated in TSI versus N (fold change = 2.3, p = 0.03) and miR-1285 is up-regulated in TSI versus TS (fold change = 4.6, p = 0.005), which was consistent with the results of the qPCR array.

**Conclusions:**

miR-147b and miR-1285 are up-regulated in inflammatory trachomatous scarring. Further investigation of the function of these miR will aid our understanding of the pathogenesis of trachoma.

## Author Summary

Trachoma is a debilitating disease that affects 40 million people worldwide. It can cause progressive fibrosis of the upper eyelid and blindness, yet the mechanism is poorly understood. We have investigated the expression of short sequences of genetic material (microRNA) that regulate gene expression. We screened for the expression of 754 microRNA sequences (miR) in genetic material isolated from conjunctival swab samples from individuals in trachoma-endemic communities in The Gambia. This sample included healthy controls, individuals with trachomatous scarring and individuals with trachomatous scarring in the presence of clinically significant inflammation. We found 82 miR that were differentially expressed. Computer simulations predict that these miR regulate genes in epithelial cell differentiation, inflammation and fibrosis pathways, all of which are involved in the scarring process. We then validated the expression of seven of these differentially expressed miR in a second larger biological sample set from The Gambia. We confirmed that miR-147b and miR-1285 have increased expression in individuals with trachomatous scarring in the presence of clinically significant inflammation. Further investigation into the functions of these miR will aid our understanding of this disease and present opportunities to develop treatments for ocular fibrotic diseases.

## Introduction

*Chlamydia trachomatis* (*Ct*) is the causative agent of trachoma, the leading cause of blindness that results from infection. Forty million people have active trachoma and eight million people suffer with unoperated trichiasis [1]. Repeated infection of the conjunctiva by this intracellular bacterium during childhood causes a chronic inflammatory response, leading to progressive fibrosis and scarring in adult life. Scarring distorts the conjunctiva and the eyelashes are pulled inward to the extent that they scratch the cornea (trichiasis), causing pain and eventually blindness.

Chronic trachomatous inflammation is known to continue in the absence of current *Ct* infection and is believed to drive the continued scarring process, however the mechanisms by which this occurs are not completely understood [2]. Messenger RNA (mRNA) expression profiling of each clinical stage of trachoma has revealed many thousands of mRNAs that are differentially expressed [2], [3]. Key pathways that are differentially regulated in the conjunctiva are innate inflammatory pathways and extracellular matrix modifiers. MicroRNAs (miR) are known to have significant roles in the regulation of inflammation, fibrosis and cell differentiation [4]–[7] and can be dysregulated upon bacterial infection [8], [9].

miR are post-transcriptional regulators of gene expression. They are single-stranded RNA molecules typically 18–22 nucleotides in length. miR bind to complementary mRNA sequences in association with the RNA-induced silencing complex (RISC), causing transcriptional degradation of the transcript or repression of its translation [10]. The seed sequence (5′ nucleotides 2–7) of the miR guides target selection [11]. Complementary target sequence sites are usually, though not exclusively, found in the 3′ untranslated region (UTR) of mRNA transcripts. For a given miR complementary sequence sites may be present on a few or several hundred different mRNA targets, indicating the potential for a few miRs to regulate complete biological processes. Indeed, a relatively small total number of miR are thought to regulate over a third of all protein-coding genes [12]. miR have profound roles in the regulation of many biological processes and interest in their various functions in health and disease is growing. The number of known mature miR (http://www.mirbase.org/) is increasing rapidly as research in this area quickly unravels miR biology.

The ability of miR to regulate entire pathways offers investigators an opportunity to reduce the complexity of the trachoma transcriptome [2]. We suggest that the differential regulation of just a few miR in trachomatous disease may underlie the substantial differences in mRNA expression that characterize each phenotypic trachoma group. This reduction in complexity will enable more targeted research into the mechanisms of disease and may identify new potential therapeutic approaches.

## Methods

### Ethical permission, study participants and clinical assessment

The study was conducted in accordance with the tenets of the Declaration of Helsinki. The Ethics Committee of the Gambian Government/Medical Research Council Unit, and the ethics committee of the London School of Hygiene and Tropical Medicine approved the study. Samples were drawn from an archive built up under the MRC study numbers SCC729, SCC1177 and SCC1274 with specific approval for miR gene expression studies under SCC L2011.03. The samples described in this paper were collected from individuals recruited in trachoma-endemic communities across the whole geographic range of The Gambia, West Africa. Written informed consent was taken from individuals at the time of sample collection. For those participants aged <16 years that wished to take part in the study consent was obtained from a parent/guardian. All samples were anonymized. Cases of trachomatous scarring (TS) were identified from screening records, community ophthalmic nurse referral and opportunistic rapid screening. Control individuals with normal conjunctivae were selected by matching for age, sex, ethnicity and location. Clinical phenotypes were assessed in the field by experienced field supervisors trained and regularly assessed in trachoma grading. FPC scores [13] were assigned and grades were agreed by two experienced trachoma physicians using high-resolution photographic records taken in the field using a Nikon D3000 SLR camera with a VR AF-S micro Nikkor 105 mm 1:2.8G ED lens. Photographs were taken at the time of sample collection. Individuals were grouped into the following clinical phenotypes for analysis: individuals with trachomatous scarring (TS) had a C score between 1–3 (mild to severe scarring) and a P score of 0 or 1 (none or mild inflammation), individuals with trachomatous scarring in the presence of clinically significant inflammation (TSI) had a C score of 1–3 and a P score of 2 or 3 (moderate to severe inflammation), and control samples from individuals with normal healthy conjunctivae (N) had no conjunctival scarring (C0), papillary inflammation (P0) or follicles (F0).

### Sample collection and processing

Swabs were taken from the upper tarsal conjunctiva using Dacron polyester-tipped swabs (Hardwood Products Company) and stored in 250 µl RNA*later* (Ambion, Life Technologies) on ice blocks in the field and then archived at −20°C until processed.

### Taqman microfluidic array miR genecards

A total of 63 specimens from the archive were selected for miR expression array profiling. Specimens were selected as representative examples of each phenotype group using the FPC scores. As control samples for these experiments, individuals with normal conjunctivae matched on age, sex, ethnicity and location were selected. MiR was extracted from swabs using the Qiagen Allprep DNA/RNA/protein kits with a modification to collect small non-coding RNAs. DNase1 digestion (Qiagen) was included. Total RNA purity was assessed by spectrophotometry using a nanodrop ND-1000 (Thermo Fisher Scientific). Reverse transcription and pre-amplification were performed using Megaplex human primer pools Av2.1 and Bv3.0 following the manufacturer's instructions (Taqman, Life Technologies). Quantitative PCR was performed using 72 µl of pre-amplified cDNA as template in the PCR master mix for the TaqMan Array Human MicroRNA genecards (Av2.0 and Bv3.0). Thermal cycling was performed on a 7900HT thermal cycler (Life Technologies). Plates were held at 50°C for 2 minutes, 94.5°C for 10 minutes, then underwent 40 cycles of 97°C for 30 seconds and one minute at 59.7°C. A total of 754 of the most well characterised unique human miR from Sanger miRBase V.14 (www.mirbase.org/) were screened. Sanger miRBase V.14 was the latest version of the miR database at the time of screening.

### Array analysis

qPCR cycle threshold (C_T_) values were processed in SDS RQ manager (Life technologies); the threshold was set to 0.05 and baselines were detected automatically. Data from each array were uploaded and analysed using the High Throughput qPCR Package (HTqPCR) in Bioconductor R (www.bioconductor.org, www.r-project.org) [14]. Sample profiles were excluded from the analysis when the median C_T_ value for the array was 40 since the majority of the C_T_ values were either close to threshold or undetermined. Individual miR were retained in the analysis only when expressed (C_T_ value<40) by at least five specimens. A and B genecard data were analysed separately due to differences in specimen performance on each card. Data were normalized to reduce technical bias in the analysis by a number of different standard methods (supplementary figure S1A & B). The coefficient of variation, standard deviation and correlation of raw against normalized data were used to evaluate the suitability of each method of normalisation, as described in Deo *et al.* (2011) [15]. The ‘Norm rank invariant’ method was chosen as the most effective normalisation strategy for both A and B cards (supplementary figure S1A & B). The distribution of the raw and Norm rank invariant normalized C_T_ values are shown in supplementary figure S2 A–D. Differential expression was then assessed by empirical Bayes/moderated t-tests using HTqPCR in Bioconductor R. These data are deposited within the NCBI GEO public database (GSE37717) and, in line with MIQE guidelines [16], details are included as supplementary table S1.

### MiR abundance

Relative abundance of miR in the conjunctiva was calculated from array C_T_ data. An average was taken of the normalized C_T_ values for all specimens (including all phenotypes) for each miR on A and B cards. The general equation for estimating relative differences in PCR was then applied to these values: 2^−(CT_target−CT_calibrator)^ where the calibrator was the most abundant miR (miR-1274B). For each miR this value was then divided by the sum of all these values to create a relative abundance.

### Network analysis

A network graph based on the specimen-to-specimen Pearson correlation was generated Biolayout express 3D v2.2 (www.biolayout.org/) [17]. The overall miR expression correlation matrix and graph were constructed from the rank invariant normalized raw C_T_ data. The graph was arranged according to patient clinical classification (N, TS, TSI). Pearson correlation coefficients (*r*)>0.7 were retained and used as cut-offs in network construction. Nodes in the graph are individual miR linked by an edge if *r*>0.7. The graph was then clustered using a Markov Clustering algorithm using default inflation values. The partitioned clusters of expression contain sets of miR that exhibit a very strong degree of co-expression across the sample. Cluster content is independent of differential expression level. The co-expression clusters were then investigated *in silico* at the individual and pathway levels.

### Pathway analysis

miR that were differentially expressed in the arrays at a significance level of p<0.05 and with a fold change (FC) over three (up or down-regulated) were entered into pathway analysis. DIANA-microT v4.0 (Beta version) target prediction was used in the DIANA mirPath software [18]. Multiple miRNA analysis was used for the significant miR within each comparison group.

### Extraction of miR for quantitative real-time PCR

Total RNA including miR was extracted from swabs using a Qiagen miRNeasy kit, incorporating a DNase1 digestion step. Total RNA purity was assessed by spectrophotometry using a nanodrop ND-1000 (Thermo Fisher Scientific). miR was reverse transcribed using miScript II RT kit with the hiFlex buffer as per the manufacturer's instructions (Qiagen). qPCR was carried out using miScript Primer Assays and the miScript SYBR Green PCR kit (Qiagen) on a 7900HT thermal cycler (Life Technologies). Ten microlitres of RT product was diluted in 100 µl H_2_0 and 0.5 µl was used as template in each qPCR assay, with 0.5 µl specific miR forward primer assay, 1 µl water, 0.5 µl universal reverse primer and 2.5 µl SYBR green master mix, in a total reaction volume of 5 µl. Each assay was performed in quadruplicate, including no template controls for each miR and for each specimen. Cycling conditions were as follows: 15 minutes at 95°C, followed by 70 cycles of 15 seconds at 94°C, 30 seconds at 55°C, and 30 seconds at 70°C. Data were collected at 94°C and 70°C. qPCR was run for 70 cycles to minimize the number of undetermined values. Fifteen percent of all miR assays had a C_T_ value over 40.

### qPCR analysis

C_T_ values were derived in SDS RQ manager (ABI, Life technologies), with a threshold of 0.05 and an automatic baseline. Four replicate tests were used to calculate the geometric mean after outliers were removed. Analysis was done in R. Specimens were removed from the analysis if the endogenous control snoU6 (U6) C_T_ values were ≥2× the standard deviation (s.d.) of the mean of all U6 C_T_s in the sample. For analysis purposes, any assay that did not amplify by 70 cycles was assigned a C_T_ value of 80 (19 assays out of a total 1552). Target C_T_ values for each specimen were normalized to U6 using Δ C_T_ = C_T__target_−C_T__U6_. For each miR within each phenotype group, the Shapiro-Wilk method was used to test for normality of distribution in the raw-data. For each comparison (N v TS, N v TSI, TS v TSI) we calculated the fold change in miR expression between the phenotypic groups, using 2^−ΔCT(median phenotype 1) −ΔCT(median phenotype 2)^. We used the Wilcoxon rank sum test (with continuity correction) to test for differences in the expression of each miR between phenotypic groups as the majority of the data were not normally distributed. Details of the qPCR and analysis in line with MIQE are included in supplementary table S1.

## Results

### Array samples

Sixty-three specimens were tested by miRNA array cards. Twenty-three specimens were excluded from A genecard profiles and 34 specimens from B genecard profiles. Basic demographic and clinical phenotype data are shown in table 1 both before and after filtering and these show that there was no systematic loss of any specific sample type based on clinical phenotype, age or sex as a result of the filtering process.

**Table 1:** Sample demographic details before and after quality control exclusion for full array analysis.

All samples before filtering	N (n = 23)	TS (n = 17)	TSI (n = 23)
Male [Female]	7 [16]	4 [13]	7 [16]
Mean age (min-max)	44.58 (3–78)	42.59 (8–75)	50.31 (3–85)
Samples included following A genecard quality control exclusion	n = 16	n = 15	n = 9
Male [Female]	5 [11]	4 [11]	2 [7]
Mean age (min-max)	48 (8–78)	43.8 (8–75)	44.9 (4–80)
Papillary hypertrophy			
0	16	14	0
1	0	1	0
2	0	0	3
3	0	0	6
Conjunctival scarring			
0	16	0	0
1	0	0	0
2	0	13	9
3	0	2	0
Samples included following B genecard quality control exclusion	n = 10	n = 12	n = 7
Male [Female]	3 [7]	4 [8]	1 [6]
Mean age (min-max)	43 (8–70)	44.5 (8–75)	41.9 (4–80)
Papillary hypertrophy			
0	10	11	0
1	0	1	0
2	0	0	2
3	0	0	5
Conjunctival scarring			
0	10	0	0
1	0	0	0
2	0	11	7
3	0	1	0

FPC grading scores (0–3) are shown for each phenotypic group.

Footnote: Age ranges between phenotypic groups are not significantly different within and between A and B genecard groups (Wilcoxon test p>0.05). Fewer B genecards were passed filtering as these cards were designed to cover less abundant miR.

### Basic characteristics of miR expression in the conjunctiva: Abundance and co-expression analysis

**Figure 1:**
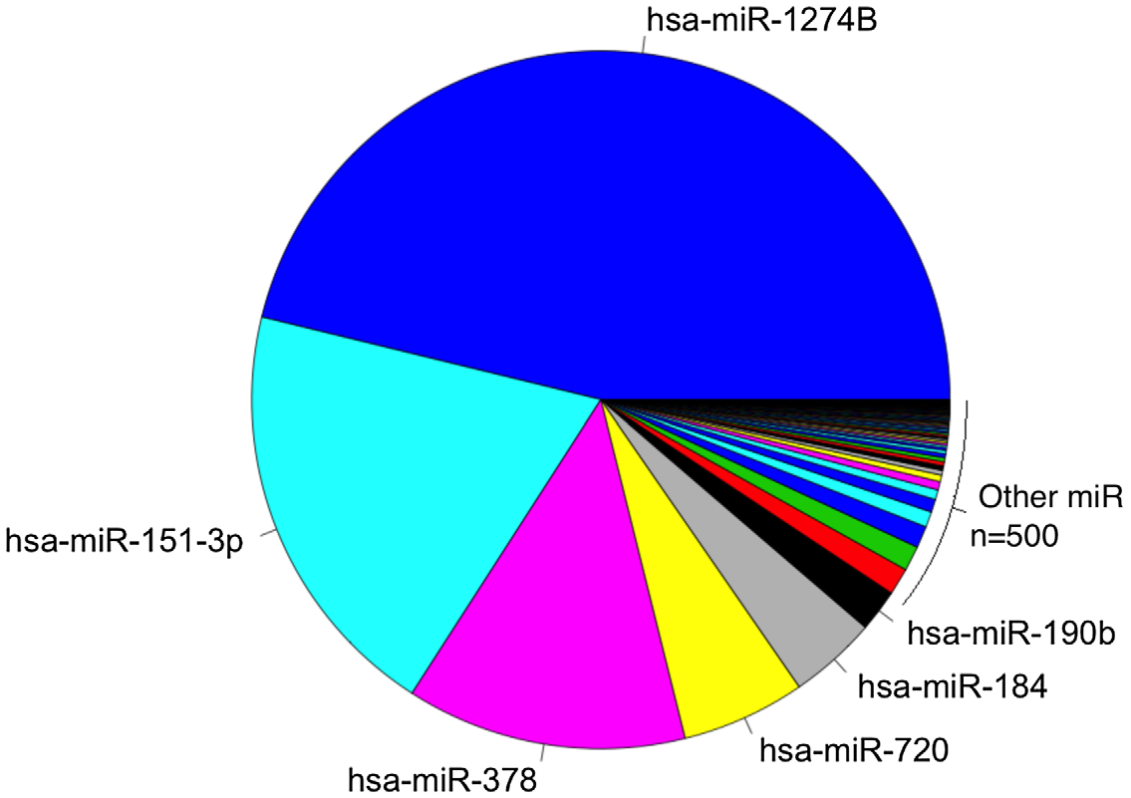
Relative abundance of miR in the conjunctiva. Abundance of all miR tested expressed relative to miR-1274B. Abundance was calculated from cycle threshold values irrespective of sample phenotype.

**Figure 2:**
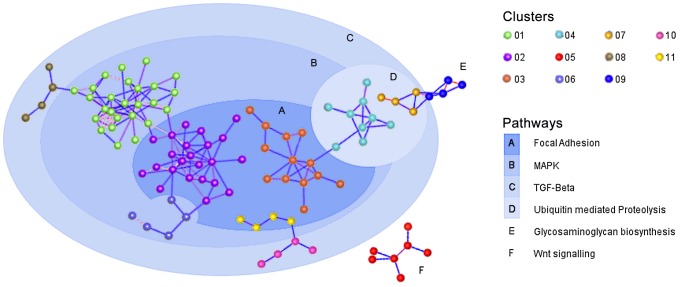
Network co-expression analysis. Clusters of co-expressed miR are shown, calculated from normalized array data irrespective of phenotype. Clusters are highlighted according to the pathway most enriched upon pathway analysis of miR in each cluster.

Following the filtering procedures described, 506/754 miRs were included in the final analysis. Relative abundance of all of 506 expressed miR in the conjunctiva was calculated from the C_T_ data. Of the miR that were tested, just six constitute 90% of total miR present in the conjunctival samples (figure 1). miR-1274B has the highest overall expression and miR-623 the lowest. Networks of co-expression, independent of differential expression, based on rank invariant C_T_ values were explored in the entire data set of 506 miR using Biolayout express 3D. The undirected graph contained 126 miR connected by 215 edges. Markov clustering partitioned the network into 11 clusters of co-expressed miR. These clusters ranged in size from 23 to 4 co-expressed miR and accounted for 80 miR in the original network. Each of the 11 clusters is laid out in figure 2 and the specific miR content of each cluster is available in supplementary table S2. The major biology revealed by these co-expression clusters indicates that these miR target mRNA in four major pathways. These are repeatedly identified and are shown in figure 2. The MAPK signaling pathway and focal adhesion pathway contain the largest number of miR target genes and have the highest levels of enrichment (over-representation).

### Differential expression analysis

A total of 82 miR are differentially expressed across the comparison groups (FC>3, p<0.05) (supplementary table S3). The number of up- and down-regulated miR in each comparison is shown in table 2. A miR that is up-regulated in the N v TS comparison has a lower C_T_ value in TS relative to N. The same applies to N v TSI and TS v TSI, where the latter is up- or down-regulated relative to the former in each comparison. A larger number of miR are differentially expressed in comparisons with the TSI phenotype. Fewer miR are down-regulated than up-regulated, particularly in the N v TSI group.

**Table 2:** Number of differentially expressed miR in array results.

	N v TS	N v TSI	TS v TSI	Total
**FC>3 p<0.05** *	**7**	**35**	**40**	**82**
*Up*	*3*	*22*	*21*	*46*
*Down*	*4*	*13*	*19*	*36*
**FC>3 p<0.01** *	**5**	**12**	**19**	**36**
*Up*	*2*	*10*	*13*	*25*
*Down*	*3*	*2*	*6*	*11*

* p-values were not adjusted or controlled by false discovery rate. We calculated 1518 independent tests of significance on the entire array data set and estimate that this would result in a false positivity rate of 50% when accepting unadjusted p>0.05.

**Figure 3:**
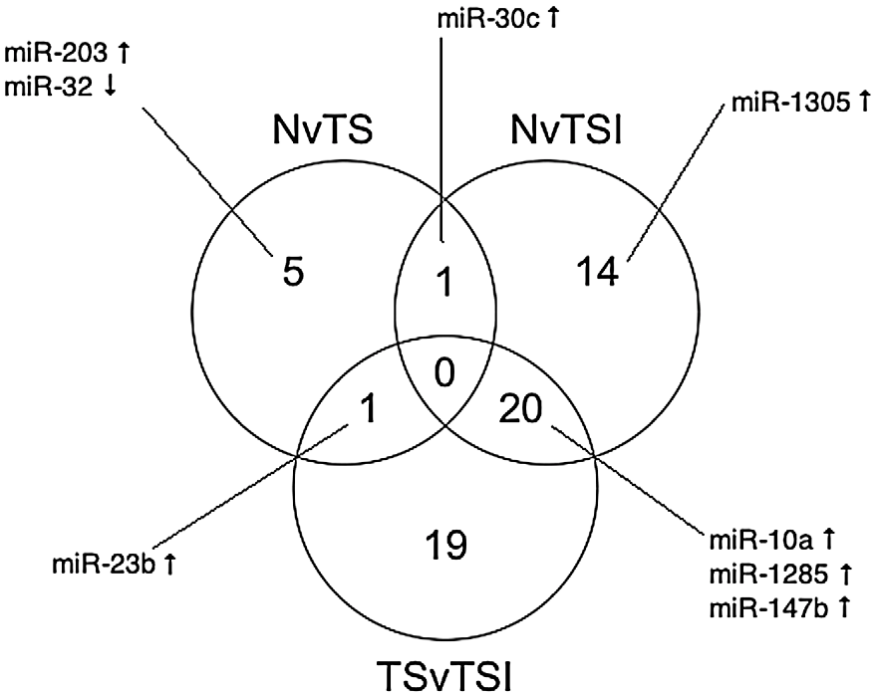
Venn diagram of differentially expressed miR. Venn diagram showing the number of differentially expressed (FC>3, p<0.05) miR that are unique or that overlap between the different clinical phenotypes. Selected miR of interest are shown with arrows illustrating whether they are up- or down-regulated in the indicated comparison group. An upward facing arrow indicates up-regulation and a downward facing arrow indicates down-regulation. * miR-23b is up-regulated in N v TS but down-regulated in the TS v TSI comparison group.

Twenty miR are differentially expressed in both N v TSI and TS v TSI comparisons (figure 3) indicating they might be features of inflammation. In contrast, very few miR are shared with the N v TS miR gene set and there are none that overlap between all three groups. This indicates that TSI and TS phenotypes are distinct and have characteristic miR signatures.

Of the 103 miR found in the networks by Markov clustering, 15 had some evidence of differential regulation based on p-value alone. miR-492 and miR-548d were both up-regulated (4.9 and 3.2 times respectively) in TSI individuals compared to controls whilst 3 miR (miR-508, miR-509 and miR-664) were >3 fold down-regulated. The miR precursor let-7b showed modest evidence of differential up-regulation in TS individuals versus normal controls (p = 0.037).

### Pathway analysis

**Figure 4:**
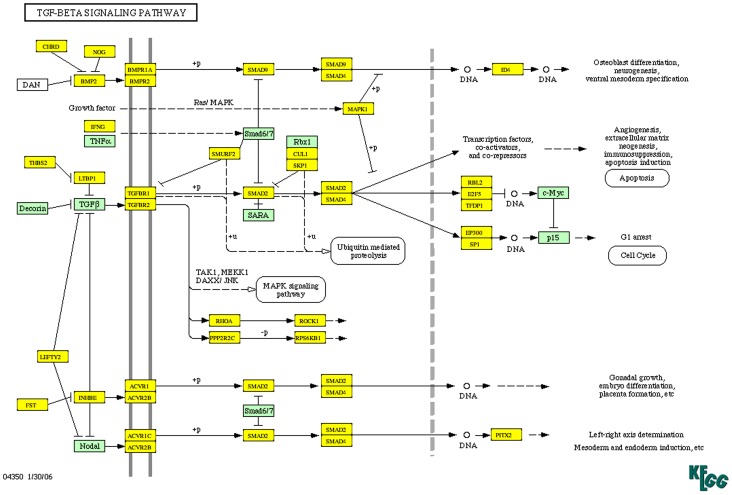
TGF-β signaling pathway. Genes highlighted in yellow are predicted targets of miR differentially regulated in N v TSI (35 miR) (FC>3 p<0.05). Genes highlighted in green are not predicted targets.

Differentially expressed miR in each comparison group (FC>3 p<0.05) were entered into DIANA mirPath pathway analysis. The top ten most enriched pathways for each comparison are listed in table 3, where a greater −ln(p-value) reflects increasing enrichment of miR targets within a pathway. Many of the same pathways are enriched in each comparison group, despite little overlap in miR between the N v TS, N v TSI and TS v TSI groups (figure 3). Axon guidance, focal adhesion, and the TGF-β signaling pathway are all present in all three groups. A large number of genes in the TGF-β pathway are predicted targets of differentially expressed miR in the N v TSI comparison (figure 4). Within the TGF-β pathway, 53% of transcripts are differentially regulated based on differences found in a mRNA transcriptome array using Ethiopian conjunctival samples (GSE23705) from similar phenotypic groups [2]. Analysis was also carried out on each phenotypic comparison group split into up- and down-regulated gene sets (supplementary table S5). Interestingly, TGF-β is enriched in the down-regulated gene set for each comparison group. Given that this pathway is enriched for miR targets that would be silenced and these miR are down-regulated, this would suggest an up-regulation or release of the TGF-β signaling pathway. Analysis was also performed on the 20 miR differentially expressed in both N v TSI and TS v TSI comparisons, with enrichment again in MAPK, TGF-β and *Wnt* pathways (supplementary table S6), supporting the hypothesis that these miR are characteristic of the major pathways under miR control in the conjunctiva and are perturbed by inflammation.

**Table 3:** DIANA mirPath pathway analysis on differentially expressed miR in each comparison group (p<0.05 and FC>3).

Pathway	# Target genes in pathway	−ln(p-value)
N v TS (n = 7)		
Axon guidance	52	25.39
Focal adhesion	70	24.14
Epidermal growth factor receptor (ErbB) signaling pathway	40	23.02
Renal cell carcinoma	31	17.13
Glioma	29	16.39
Tight junction	48	15.51
Non-small cell lung carcinoma	25	14.35
Regulation of actin cytoskeleton	65	14.3
Small cell lung cancer	34	14.15
TGF-β signaling pathway	35	13.93
N v TSI (n = 35)		
Focal adhesion	94	19.86
Axon guidance	66	18.49
Regulation of actin cytoskeleton	97	17.43
MAPK signaling pathway	113	17.23
TGF-β signaling pathway	51	16.93
ErbB signaling pathway	50	16.77
Ribosome	2	16.58
Ubiquitin mediated proteolysis	66	15.68
*Wnt* signaling pathway	71	14.58
Oxidative phosphorylation	11	13.61
TS v TSI (n = 40)		
Axon guidance	77	24.39
Adherens junctions	52	24.34
MAPK signaling pathway	127	18.99
Ribosome	3	18.95
*Wnt* signaling pathway	82	18.49
TGF-β signaling pathway	56	17.39
Focal adhesion	100	16.77
Oxidative phosphorylation	13	15.88
Ubiquitin mediated proteolysis	72	15.25
Renal cell carcinoma	43	13.2

### Validation of differential expression of selected miR using an alternative RT-qPCR system

In the validation set it was not feasible to assay the expression of all the potentially differentially expressed miR and we selected for follow-up a small number of miR that exhibited a high FC, low p-value and homogeneity in the raw data. Seven miR were selected for follow-up (supplementary table S7) including three miR that were differentially regulated in the N v TS comparison (miR-30c, miR-32, miR-203) and four from the N v TSI comparison (miR-10a, miR-147b, miR-1285, miR-1305). Each candidate miR was tested for differential expression in a second sample of 194 independent archival Gambian clinical specimens, selected as representative examples of each phenotype group. In these experiments, small nucleolar (sno) U6 RNA was used as the calibrator snoU6 C_T_ values were not different between the phenotypic groups, which implied that it was a stably expressed reference gene (supplementary information figure S3). Five specimens were excluded because they had outlying snoU6 values (average C_T_>2 s.d. of the sample mean), leaving a total of 189 specimens to be tested for statistical differences in expression levels. Summary statistics for these 189 specimens are shown in table 4.

**Table 4:** qPCR sample demographic summary including FPC grading scores (0–3) for each phenotypic group.

Specimen number in each group	C (n = 93)	TS (n = 74)	TSI (n = 22)
Male [Female]	23 [70]	19 [55]	8 [14]
Mean age (min-max)	51.4 (16–87)	51.7 (16–80)	47.7 (2–80)
Papillary hypertrophy score	Number of samples		
0	91	36	0
1	2	38	0
2	0	0	19
3	0	0	3
Conjunctival scarring score	Number of samples		
0	93	0	0
1	0	9	1
2	0	61	15
3	0	4	6

Footnote: Age ranges between phenotypic groups were not significantly different (Wilcoxon test p>0.05). One individual in the TS group also had a follicular grade (F) of 1, and three individuals in TSI had F3.

Data were tested for differential expression between the three phenotypic groups as is presented for the analysis of the array data (N, TS and TSI). Only miR-1285 and miR-147b showed a significant difference between the different phenotypic groups in this validation set (table 5). MiR-147b was up-regulated 2.3 fold in individuals with TSI relative to N (p = 0.0332). This is consistent with the array results in which miR-147b was up-regulated 9.6 fold in individuals with TSI versus N. MiR-1285 was up-regulated 4.6 fold in TSI relative to TS (p = 0.005). This is also consistent with the array results in which miR-1285 was up-regulated 16 fold in TSI versus TS.

**Table 5:** Results of qPCR differential expression analysis.

	Median deltaCT (IQR)			N v TS		N v TSI		TS v TSI	
miR	N	TS	TSI	Fold difference	p-value*	Fold difference	p-value*	Fold difference	p-value*
miR-10a	16.3 (14.7–18.9)	16.1 (13.7–20.4)	17.4 (14.5–20)	1.149	0.554	0.467	0.353	0.406	0.248
miR-30c	5.3 (3.5–6.7)	5 (2.8–7.2)	5.9 (3.8–7.6)	1.231	0.813	0.660	0.761	0.537	0.362
miR-32	14 (11–18.1)	14.6 (11.9–18)	15.2 (13.3–17.1)	0.660	0.403	0.435	0.332	0.660	0.845
miR-147b	6.4 (5–9.6)	6.8 (4.8–10.7)	5.2 (3.1–7.8)	0.758	0.783	2.297	0.033	3.031	0.078
miR-203	4.6 (2.2–5.1)	3.7 (1.9–5.8)	3.8 (1.2–6.3)	1.866	0.097	1.741	0.234	0.933	0.706
miR-1285	7 (5.1–8.7)	8 (5.4–9.7)	5.8 (3.8–8)	0.5	0.214	2.297	0.059	4.595	0.006
miR-1305	12.7 (11–14)	12.6 (9.3–14.6)	12.5 (10.6–13.7)	1.072	0.535	1.149	0.562	1.072	0.709

* Unadjusted p-values are presented. With no inflation of p-values the chance of finding one or more significant differences in 21 tests = 65.9%. Bonferroni's adjustment indicates critical p-value = 0.002 and assuming outcomes are moderately correlated (r = 0.5) then a critical p-value = 0.01 would be required.

## Discussion

Array analysis revealed that a large number of miR are potentially differentially regulated between different disease states and healthy controls. Trachomatous scarring with inflammation (TSI) has a distinct miR signature compared to scarring trachoma (TS). TS may be a less active disease process than TSI as fewer miR were differentially regulated. On validation, two miR remained significantly differentially regulated. MiR-147b was up-regulated in individuals with TSI compared to N and miR-1285 was up-regulated in people with TSI compared to those with TS alone. In a transcriptome analysis of similar phenotypic comparison groups in Ethiopians with scarring trachoma [2], 25% of predicted targets of miR-1285 and 52% of predicted targets of miR-147b predicted targets (TargetScan v6.2) were differentially regulated (adjusted p<0.05).

MiR-1285 directly targets the 3′UTR of p53 mRNA in HEK 293T cells [19]. The loss of p53 is associated with many cancers via disruption of the normal function of p53 in the initiation of apoptosis and growth arrest. In contrast, Hidaka and colleagues [20] find miR-1285 to be a tumor suppressor. Expression levels of miR-1285 were reduced in clinical samples of renal cell carcinoma (RCC) compared to healthy mucosa and miR-1285 transfection in RCC cell lines *in vitro* led to inhibition of cell proliferation, migration and invasion [20]. The authors verified transglutaminase 2 (TGM2) as a target of miR-1285. Interestingly, TGM2 is linked to several cancers and the process of epithelial-mesenchymal transition (EMT) [21]. EMT can be initiated by chronic inflammation [22] and is implicated in the pathology of many fibrotic diseases [23], so could have a role in trachomatous disease. The conflicting conclusions of these studies are likely due to the different cell types used. *In vitro* studies in primary conjunctival epithelia will be required to understand the function of miR-1285 in chlamydial infection and trachoma.

There is limited literature on the clinical significance of changes in miR-147b expression, but it is known to be down-regulated in rectal cancer [24]. This is consistent with the ability of miR-147b to induce apoptosis four days post-transfection in A549 cells [25]. *Mmu*-miR-147, a functional homologue of human miR-147b, is induced by multiple toll-like-receptor (TLR) signals and negatively regulates inflammation in murine macrophages [26]. It acts in a negative feedback loop to prevent excessive inflammation. Bertero and colleagues [25] showed that LPS (bacterial lipopolysaccharide) and TNFα strongly induced hsa-miR-147b expression in A549 cells *in vitro*; supporting the hypothesis that miR-147b has a homologous role in the regulation of inflammation in humans. The up-regulation of miR-147b seen in the TSI phenotypic group may reflect ongoing and uncontrolled inflammation. Understanding the role of miR-147b in TSI should also be aided by further *in vitro* functional studies.

MiR-23b-5p is up-regulated in TS relative to both N (FC = 3.6 p = 0.026) and TSI (FC = 5.8 p = 0.008) (figure 3), indicating that it might be a feature of scarring in the absence of inflammation. MiR-23b is a member of the miR-23b cluster, which includes miR-27b and miR-24-1. This cluster is known to target members of the TGF-β signaling pathway [27]. An up-regulation of miR-23b, as is seen in scarred individuals, would lead to a decrease in TGF-β signaling. Although this may seem counterintuitive, any dysregulation of the TGF-β signaling pathway could lead to aberrant wound healing [28]. MiR-23b is also anti-inflammatory through inhibition of NFkB activation [29], preventing up-regulation of inflammatory cytokines such as IL-17. In turn, IL-17 inhibits miR-23b, leading to inflammation. An up-regulation of IL-17A is associated with active trachomatous disease [30]. The relative down-regulation of miR-23b in TSI compared to TS and N conjunctival samples could reflect a down-regulation of this miR's expression by IL-17 in individuals with TSI.

MiR-30c was up-regulated 15 fold in N v TS (p = 0.01) and 11 fold in N v TSI (p = 0.04) in the microarray experiments. This miR is thought to regulate fibrinolysis and collagen production through targeting serine protease inhibitor SERPINE1 and connective tissue growth factor (CTGF) respectively [31]. Over expression of this miR is known to inhibit the proliferative and migratory properties of endometrial cancer cells [32], however qPCR validation found no association of this miR with disease.

Pathway analysis revealed that many of the same pathways are enriched amongst predicted targets of differentially expressed miR in each comparison group. This is surprising due to the minimal overlap of differentially expressed miR in N v TS compared with the other groups. Many of these pathways are characteristic of epithelial cell and fibroblast communication, differentiation and fibrosis. In particular, the *Wnt* pathway has been implicated in the disruption of epithelial cell homeostasis and the pathology of *C. trachomatis* infection [33]. Importantly, epithelial cell differentiation, development and cytoskeleton remodeling pathways (including TGF-β and *Wnt*) are enriched in gene sets from a differential expression analysis of transcriptome data of similar phenotypic groups in Ethiopians [2]. Of all the members of the KEGG defined TGF-β pathway, 53% are differentially regulated in this Ethiopian transcriptome [2]. We theorise that the miR that are differentially expressed in this study are at least partly responsible for the observed changes in the normal function of the *Wnt* and TGF-β pathways in trachoma patients.

Investigation of miR abundance in the conjunctiva shows that most miR have very low levels of expression, with just a few making up the vast majority of the population. MiR-1274B is the most highly abundant miR in the conjunctiva, but the nature of this miR has been called in to question. A previous study [34] found evidence that miR-1274B may not be a canonical miR, rather that it is a tRNA-derived small RNA (tsRNA). tsRNAs are thought to be abundant in the genome [26] and may have a role similar to miR in regulating gene functions [35]. It may be the case that the high abundance of miR-1274B can be explained simply by its origin in tRNA and the generally high abundance of tRNA in cells. Regardless of this uncertainty over its designation as either a miR or a tsRNA, a role for miR-1274B in the regulation of gene expression cannot be excluded at this time.

The discrepancy between the array results and qPCR validation could be due to a high number of false positives accepted in the initial analysis or as a result of a number of technical differences leading to differential miR isolation, extraction, amplification bias and normalisation [36]. Different methods of miRNA isolation and qPCR were employed in this study in the screening and validation stages which could introduce technical variation [37]. In addition, a non-proscriptive filtering process was used in the identification of potentially differentially expressed miR. Acceptance of a high number of potential false positive associations at this initial filtering stage was considered acceptable based on the arguments presented by Rothman [38] on principles of p-value adjustment and enabled us to explore the wider biology of miR conjunctival expression (supplementary table S4). As a result, many of the miR chosen for follow up in the validation clinical samples appear not to be differentially regulated. This highlights the need to verify array-profiling results even when the method of choice is the apparently robust gold standard technique to examine differential expression [39]–[41]. Furthermore the design of the B genecards, which cover less abundant miR, adds difficultly to analysis pipeline. Even with pre-amplification the amount of genetic material that can be obtained from a conjunctival swab is very small. Specimens were judged to perform less well on B genecards leading to the exclusion of a larger number of specimens, resulting in a reduction of the number of biological replicates in each phenotypic group and therefore some loss of statistical power. The raw and analysed data is publicly available in the NCBI GEO.

This is the first description and initial identification of specific miR expression in an ocular fibrotic disease of humans. It is possible that these miR play a role in other ocular surface inflammatory diseases. We highlight the major pathways under the control of miR that are expressed in the conjunctival epithelium and suggest that it is plausible that dysregulation of expression in these miR leads to the release of the TGF-β signaling pathway in trachomatous disease. Two miR with significantly increased expression in trachomatous scarring and inflammation were identified (miR-147b and miR-1285). In order to understand the mechanisms by which these miR may contribute to health and disease of the conjunctiva the associations of miR-147b and miR-1285 with trachomatous disease now requires further study. A combination of *in vitro* experimentation in model systems and *in vivo* application in animal models will also facilitate our understanding of this association and whether these miR are reflective or causative effectors of disease. Research in this area of RNA biology is a rapidly evolving field that is only now beginning to realize its potential. We hope that its application to trachomatous disease may lead to the development of therapeutics or biomarkers for the diagnosis and treatment of trachoma and other fibrotic ocular pathologies.

## Supporting Information

Figure S1 Supplementary figure S1A & B. Coefficient of variation (CV) for uncorrected and normalized array data for A and B card genesets. Various methods of normalization were tested (Left to right: uncorrected data, geometric mean, quantile, delta-C_T_ using RQ manager, delta-C_T_ using geNorm, norm rank invariance, scale rank invariance).
(TIFF)

Figure S2 Supplementary figure S2A–D. Boxplots of C_T_ distribution for each sample in A and B geneset groups after quality control filtering before normalization (A, B), and after normalization (C, D).
(TIFF)

Figure S3 Boxplots of endogenous control snoRNA U6 raw C_T_ distribution in each phenotype group. These distributions are not significantly different (Kruskal-Wallis p = 0.5469).
(TIFF)

Table S1 MIQE guidelines for array and qPCR processing.
(XLS)

Table S2 MiR in the 11 clusters derived from Markov chain clustering algorithm of array data. Footnote: The most enriched pathway among predicted targets of miR in each cluster is shown with values and FC for each miR from the array analysis.
(XLSX)

Table S3 Differentially regulated miR (FC>3 p<0.05) in each phenotypic comparison group in the array analysis.
(XLSX)

Table S4 Number and percentage of false positives in trachoma miR array study. Footnote: With no inflation of p-values the chance of finding one or more significant differences in 1518 tests = 100%. Bonferroni's adjustment indicates critical p-value = 3.38×10^−5^ and assuming outcomes are moderately correlated (r = 0.5) then a critical p-value = 0.001 would be required.
(XLSX)

Table S5 Pathway analysis of up- and down-regulated miR in the array analysis (FC>3 p<0.05).
(XLSX)

Table S6 Pathway analysis on 20 miR differentially expressed (FC>3 p<0.05) in both N v TSI and TS v TSI comparisons.
(XLSX)

Table S7 MiScript primer assays used in qPCR validation: accession number and mature miR sequence.
(XLSX)
