# Supplementary figures and images for: Conjunctival MicroRNA Expression in Inflammatory Trachomatous Scarring
Source: PLoS Negl Trop Dis. 2013 Mar 14;7(3):e2117. doi: 10.1371/journal.pntd.0002117 (PMC3597489; doi:10.1371/journal.pntd.0002117)

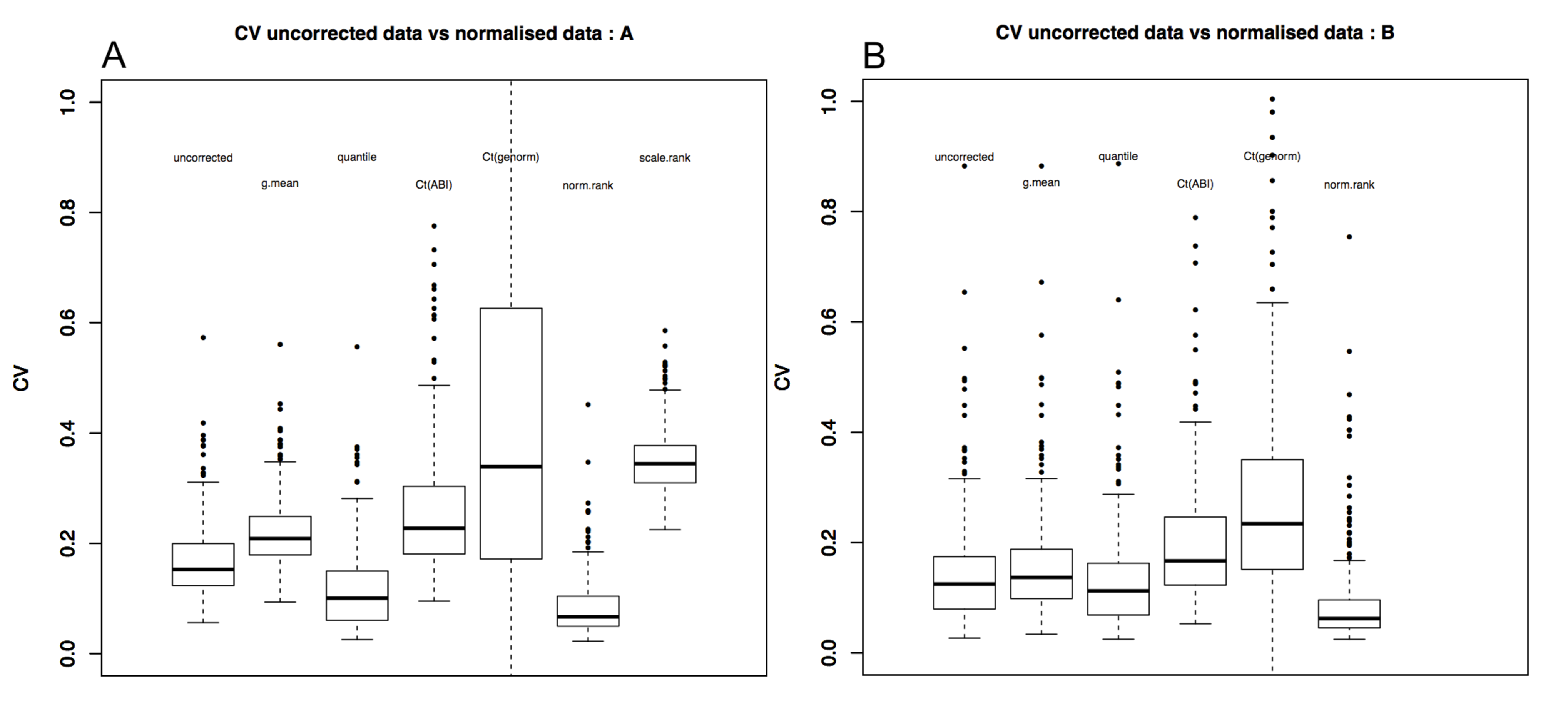

Supplement: Figure S1 — Supplementary figure S1A & B. Coefficient of variation (CV) for uncorrected and normalized array data for A and B card genesets. Various methods of normalization were tested (Left to right: uncorrected data, geometric mean, quantile, delta-CT using RQ manager, delta-CT using geNorm, norm rank invariance, scale rank invariance). (TIFF) [file pntd.0002117.s001.tiff]

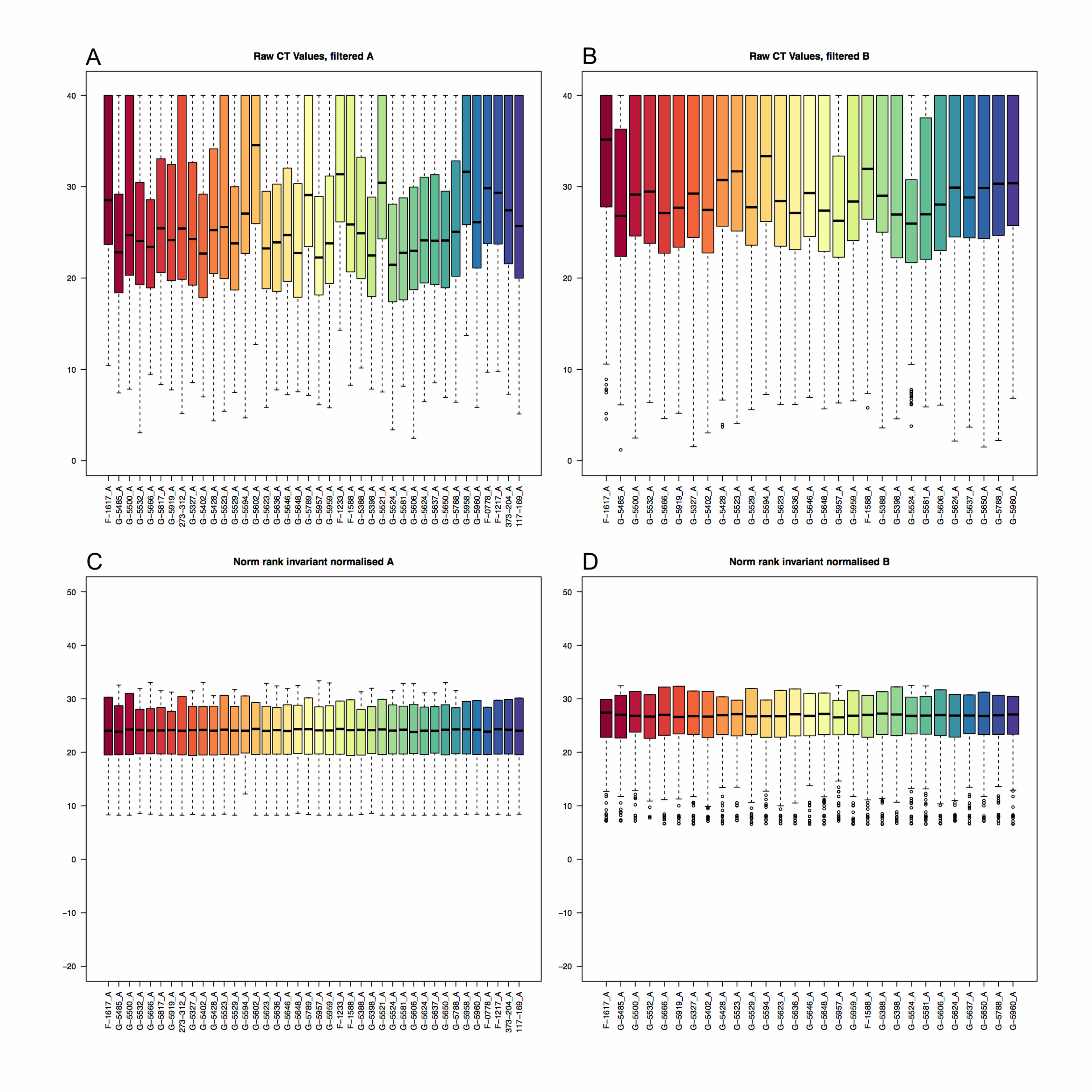

Supplement: Figure S2 — Supplementary figure S2A–D. Boxplots of CT distribution for each sample in A and B geneset groups after quality control filtering before normalization (A, B), and after normalization (C, D). (TIFF) [file pntd.0002117.s002.tiff]

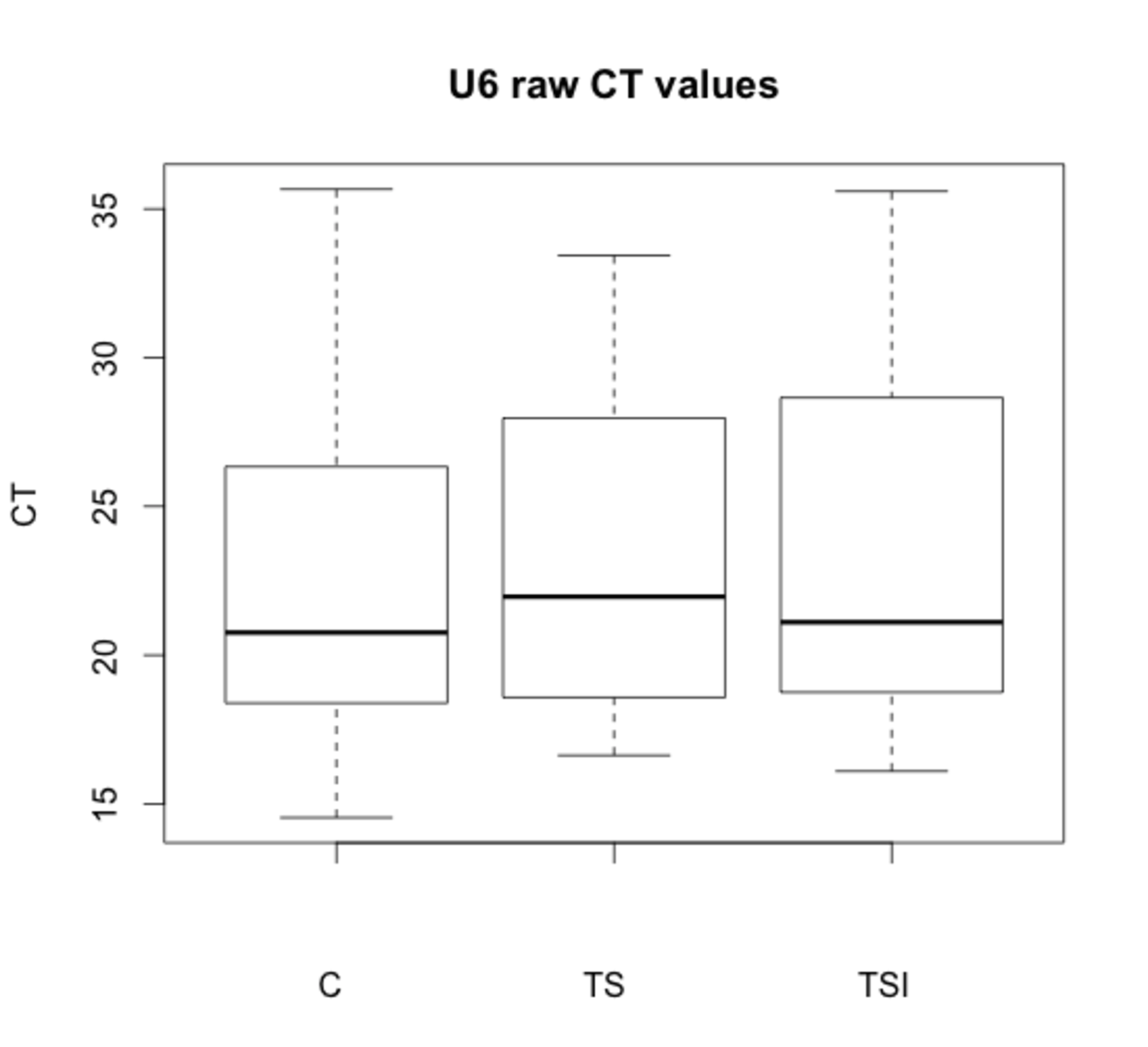

Supplement: Figure S3 — Boxplots of endogenous control snoRNA U6 raw CT distribution in each phenotype group. These distributions are not significantly different (Kruskal-Wallis p = 0.5469). (TIFF) [file pntd.0002117.s003.tiff]
